# Comparing the effects of nursing versus peer-based education methods on the preoperative anxiety in infertile women: An RCT

**DOI:** 10.18502/ijrm.v17i12.5795

**Published:** 2019-12-30

**Authors:** Farahnaz Farnia, Abbas Aflatoonian, Athareh Kalantari

**Affiliations:** ^1^ Department of Nursing, Nursing-Midwifery School, Shahid Sadoughi University of Medical Sciences, Yazd, Iran.; ^2^ Research and Clinical Center for Infertility, Yazd Reproductive Sciences Institute, Shahid Sadoughi University of Medical Sciences, Yazd, Iran.

**Keywords:** Education, Infertility, Preoperative anxiety, Nurse, Peer.

## Abstract

**Background:**

Preoperative anxiety is a common event in patients expecting surgery. Education can play an important role in reducing the negative effects of anxiety on the response to treatment. Therefore, identifying the appropriate method is important.

**Objective:**

The aim of this study was comparing the effects of nursing versus peer-based education on the preoperative anxiety in infertile women.

**Materials and Methods:**

In this clinical trial, 198 eligible infertile women were randomized into three groups (n = 66/each): the nurse-educated, peer-educated, and the controls. The Spielberger State-Trait Anxiety Inventory was filled out by all participants for measuring the patient anxiety at the time of hospital admission and prior to surgery. Participants in the nurse-educated and peer-educated groups received a group education program by a nurse or peer, respectively, after the initial completion of the Spielberger State-Trait Anxiety Inventory.

**Results:**

The mean score anxiety was 44.47, 46.92, and 42.60 at the time of hospital admission and 39.38, 41.06, and 43.42 prior to surgery in nurse-educated, peer-educated, and the control groups, respectively. There was a significant difference in the mean score of anxiety in each group before and after the intervention (p < 0.0001). However, the difference between the groups was not significant.

**Conclusion:**

Our findings demonstrate that nursing and peer education programs both reduce the preoperative anxiety. Hence, optimal use of the peer's potential regarding the compensation for staff shortage for preoperative education as well as investigating the effect of individual education is suggested for further studies.

## 1. Introduction

Anxiety is an unpleasant feeling of pressure on human actions and behavior that can be caused by being placed in a stressful process (1).

Hospital admission and waiting for surgery experience is a stressful time for all patients (2). Solgajova and colleagues believe that "gender, the type of operation and anesthesia, and time to surgery are important predictors of pre-operative anxiety” (3). Women, in general, experience a higher level of anxiety than men (4).

Mood disorders and anxiety in women are probably related to the level of estrogen and progesterone (5). Besides, women who have undergone infertility treatment have experienced an equal or more excitement levels than those with cancer or cardiac disease (6). In vitro fertilization (IVF) is a high-cost process with low chances of success. Therefore, 80 % of patients report moderate to severe levels of anxiety (7). The duration of treatment is also a stressful factor for infertile couples.

As such, the identification of effective approaches to reducing anxiety at this time is deemed necessary (8). These mental stresses can be a threat to IVF treatment outcomes (9). On the other hand, preoperative anxiety, as a challenging concept in the patients who are candidates for surgery, has always been important (10). In this regard, different methods such as therapeutic relationship, patient's education, and the prescribing medications like beta blockers have been advised to reduce preoperative anxiety (11). Some studies have indicated the positive effect of preoperative education on the patients' anxiety levels (12, 13); while in Sjoling and colleagues study, they did not obtain this result (14).

In the meanwhile, Demarco and colleagues believe that patient education should be considered as a part of the healthcare program (15).

For this purpose, medical team, especially nurses, by assessing the psychological problems in patients during treatment steps and using the anxiety-reducing methods such as education can play the important role in reducing anxiety level (16).

Recently, another method under the title of peer education has caught researchers' attention. It is a process in which motivated and educated individuals take the responsibility of organized or conversational education of peers (17).

However, the impact and preference of nursing and peer education methods on the preoperative anxiety in comparison to each other have not been indicated. Due to the high level of preoperative anxiety in infertile women who underwent surgery along with the shortage of nursing staff and time for communication with patients and educating them, it seems that the use of peers can be helpful and beneficial (18).

Therefore, this study was conducted with the aim of “comparing the effects of nursing versus peer-based education on the preoperative anxiety in infertile women.”

## 2. Materials and Methods

In this clinical trial, 198 eligible infertile women who were candidates for ovarian puncture referred to the Research and Clinical Center for Infertility, Yazd, Iran (from 2016 to 2017) were randomized into three equal groups (n = 66/each): the nurse-educated, the peer-educated, and the controls. Our inclusion criteria were as follows: age between 18 and 45 yr, knowledge of Persian language, and literate. Women with mental illness, drug addiction, cancer, and those taking anti-anxiety medications were excluded.

The simple random sampling was carried out by the use of random allocation to each of the three groups. By considering α = 0.05, ß = 0.02 (19), and 10% of fall-off, the sample size was determined as 70 women for each group.

Participants in the nurse-educated and peer-educated groups received a group education program by a nurse or a peer, respectively, after the initial completion of the Spielberger State-Trait Anxiety Inventory (STAI).

The nurse and peer-educated groups were on 30-60 min group education program after the completion of the primary questionnaire. However, the control group was only educated at the time of their discharge, which is part of the section's routines. The educational package contained the type and duration of surgery, postoperative cares, including diet, medicine consumption, the quality and amount of rest after surgery, and postoperative activity.

During this education, the nurse and the peer were allowed to express positive experiences. To educate the peer group, three qualified peers were selected according to the following criteria: history of successful ovarian puncture, high school diploma or higher, and having low levels of anxiety based on the STAI. These selected peers were educated before sampling by the nurse educator of another group about their roles in the study, the importance of instructing patients, and the educational content in two sessions. Then, through an experimental instruction, one of the peers was selected as the educator for the peer-educated group. She was a 24-yr-old housewife and a resident of Yazd who had a high school diploma and a child from IVF.

A two-part questionnaire, including demographic information and the Spielberger inventory (a 20-item standard questionnaire for obvious anxiety) was used for data collection. Each of the 20 items was a statement and a scale between 1 and 4 were allocated based on the given answer. The score of four in ten statements indicated high levels of anxiety; and conversely in items 1, 2, 5, 8, 10, 11, 15, 16, 19, and 20 indicated no anxiety. The STAI questionnaire was scored from 20 to a maximum 80 points (20).

The reliability and validity of the questionnaire have been investigated in prior studies (20, 21). The questionnaire was filled out in a self-report manner in the researcher's presence at the time of admission (two days before the operation) and immediately after the intervention (prior to surgery).

### Ethical consideration 

This research proposal was approved by the Ethics Committee of Research and Clinical Center for Infertility, Yazd, Iran (Code: 289668). After obtaining written informed consent from all participants, the anxiety was quantified using the STAI at the time of hospital admission and prior to surgery.

### Statistical analysis

Data analysis was conducted using SPSS (Statistical Package for the Social Sciences, version 19.0, SPSS Inc., Chicago, Illinois, USA) software. Data were represented as mean ± standard deviation and absolute and relative frequencies. Paired *t*-test and ANOVA test were performed to examine the significant differences between the groups at the 0.05 level (p < 0.05).

## 3. Results

Totally, 240 women candidates for ovarian puncture enrolled in this study. 3 women due to unwillingness to continue cooperation and incomplete questionnaire and 9 women in the follow up step excluded. Finally, data analysis was conducted on 198 participants in three groups (n = 66/each) (Figure I). The majority of the participants (63.3%) were 25 to 34 yr old, 38.9% of them were married (1 to 5 yr), and 51.5% had less than five years of infertility history. Three groups were matched for age, marriage duration, and history of infertility (Table I). According to the results, the mean ± SD of the admission anxiety score in the nurse-educated, the peer-educated, and the control groups were 44.47 ± 11.12, 46.92 ± 9.87, and 42.60 ± 10.36, respectively, and the mean anxiety score immediately prior to the surgery were 39.38 ± 11.08, 41.06 ± 9.27, and 43.42 ± 10.61, respectively (Table II).

There was a statistically significant difference between nurse-educated and peer-educated groups in the mean of preoperative anxiety score (p < 0.0001). However, no significant difference was observed in the control group (p = 0.37). In addition, the comparison of anxiety score between the groups did not show any significant difference in each of the studied periods (Table II, Figure 2).

**Table 1:** Demographic characteristics in three study groups (n = 66/each)


**Variables**	**Groups**	**Nurse-educated**	**Peer-educated**	**Control**	**Total N = 198**	P-value^*^
	< 25	7 (10.6)	10 (15.2)	5 (7.6)	22 (11.1)	
	25-34	40 (60.6)	39 (59.1)	47 (71.2)	126 (63.6)	
<brow>-3</erow> Age (Yr)	≥ 34	19 (28.8)	17 (25.8)	14 (21.2)	50 (25.3)	<brow>-3</erow> 0.49
	1-5	28 (42.4)	26 (39.4)	23 (34.8)	77 (38.9)	
	6-10	25 (37.9)	27 (40.9)	22 (33.3)	74 (37.4)	
	11-15	8 (12.1)	7 (10.6)	18 (27.3)	33 (16.7)	
<brow>-4</erow> Duration of marriage (Yr)	> 15	5 (7.6)	6 (9.1)	3 (4.5)	14 (7.1)	<brow>-4</erow> 0.19
	1-5	37 (56.1)	33 (50)	32 (48.5)	102 (51.5)	
	6-10	19 (28.8)	24 (36.4)	16 (24.2)	59 (29.8)	
	11-15	9 (13.6)	6 (9.1)	15 (22.7)	30 (15.2)	
<brow>-4</erow> Infertility duration (Yr)	> 15	1 (1.5)	3 (4.5)	3 (4.5)	7 (3.5)	<brow>-4</erow> 0.28
Data presented as n (%), ^*^ANOVA test						

**Table 2:** Comparison of anxiety score in admission time and prior to the surgery between three study groups


**Groups**	**Anxiety score**		P-value**
	**In admission time**	**Prior to the surgery**	
Nurse-educated	44.47 ± 11.12	39.38 ± 11.08	< 0.001
Peer-educated	46.92 ± 9.87	41.06 ± 9.27	< 0.001
Control	42.60 ± 10.36	43.42 ± 10.61	0.37
Total	44.66 ± 10.58	41.29 ± 10.42	< 0.001
P-value*	0.06	0.08	
Data presented as Mean ± SD, *ANOVA test; **Paired t-test			

**Figure 1:**
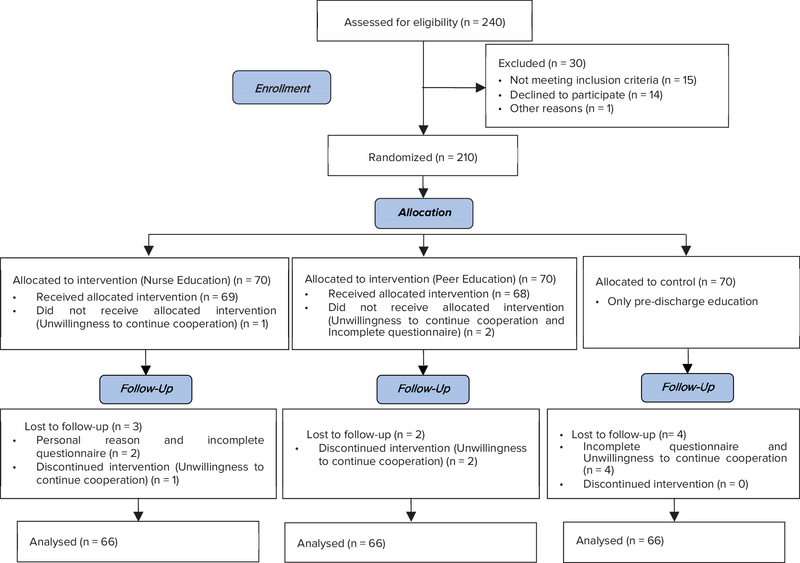
Consort flow diagram.

**Figure 2:**
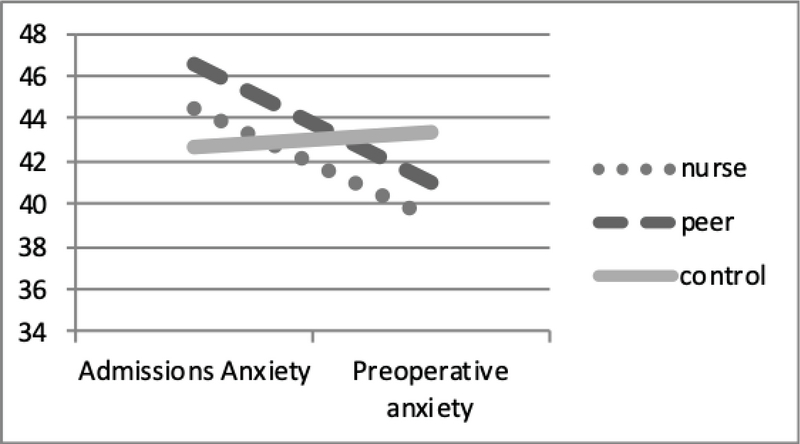
Comparison of anxiety score in admission time and prior to the surgery between the three study groups.

## 4. Discussion

This study examined and compared the effect of preoperative educational approaches by a nurse and peer on the women candidate for ovarian puncture surgery. Although both education methods were effective in reducing patients' immediate preoperative anxiety, results show that nurse-education seems to be more effective than peer-education. After education, the anxiety levels of both education groups also reduced significantly. Comparison of the mean anxiety score at two investigated times was statistically significant for each group. In this regards, the anxiety scores of the nurse-educated and the peer-educated groups were decreasing in comparison to the control group. These results are consistent with Mohammad Pourhadaki's study (22). In the current study, the control group received only the routine care (discharge educating) and had higher level of anxiety than at admission. This finding highlights the importance of including preoperative education for patients in the care protocol.

Our findings were in line with those reported by the previous studies (1, 13, 23). According to the results of this study, although the control group had a lower anxiety score at the time of admission compared to the other two groups, the three groups under the investigation were not different statistically. This finding is in accordance with the previous results (22, 24). Nelson and colleagues in their research showed that patients' fear and anxiety would be reduced by giving pre-surgery information, and all of patients who received pre-surgery information said they had received useful information; 86% felt that their anxiety had decreased (25). In addition, Pfeiffer and Cristopherson in a study concluded that there was no significant difference between the average anxiety score of those who received written educational content prior to admission and those who received the education the day before the surgery (26). Unlike the results of the present study, some previous studies reported the non-effect of preoperative education (27). However, the results of the present study may prove the positive effects of preoperative education on patients' anxiety. In other words, nurse-educated and even peer-led education can both have similar and positive effects on patients' overall health.

In addition, the mean preoperative anxiety score in the nurse-educated group was lower than the Error! Not a valid link. Two other groups. This finding indicates the importance of nurses' role in assessing patients' psychological problems during the treatment steps and using methods such as patient education that reduces their anxiety. Zhang and co-workers believe that education and counseling by nurses before surgery reduced preoperative anxiety in the patients undergoing open cardiac surgery (28). However, Asilioglu and colleagues did not find this effect in the same group of patients in Turkey (27). Evidence show that factors like age, gender, history of surgery, familiarity with non-pharmacological methods, the type, and site of surgery, as well as the culture can affect the levels of anxiety (3, 4, 27). Therefore, cultural differences and the type of operation should explain the difference in results from the present study with ones carried out in other countries.

The comparison of anxiety scores in the peer-education group at two investigated times also yielded a significant correlation. In line with the present study, Kumakech *et al*. revealed a reduction in social mental distress, particularly depression, stress, anxiety, and anger, in the experimental group after the interference of the peer group (29). In this way, most studies that have been used by the peer group as an educational approach, especially on the psychological symptoms of patients, indicate the positive impact of this educational method. The positive impact of peer education may be due to the fact that patients are more likely to be confident in the experiences and information of people who have had the same conditions as those who are successful in controlling their symptoms and problems and try to use methods to control their illness that other patients with same illness have benefited from and have managed to overcome their problems.

The peer in the present study attempted to simply and effectively convey their experiences to the patients. Therefore, this willingness plus sufficient sample size, random allocation, and patient's cooperation in conveying experiences can be the strength of this study in comparison with the previous studies. This finding indicates that peer education can be effective in reducing the patients' anxiety. Most studies in the peer group as an educational approach, especially on psychological symptoms of the disease, have noted the impact of these education methods (19, 22, 23).

In addition, it is probable that the reduction of anxiety in the two educated groups is caused by the establishment of the relationship and the reassurance of trust in the patients waiting for surgery. In this regard, Farnia *et al*., in a qualitative study, described the relationship between nurses and patients in the operating room in two ways: "understanding threats and risks" and "feeling of need for assurance" as the underlying factors for communicating with patients who were candidates for the surgery (2).

In contrast to these findings, Ortiz and coworkers concluded that the educational pamphlets may increase patient satisfaction with the recognition of the surgical procedure but do not reduce the preoperative anxiety (30). The probability of obtaining information about the treatment process by participants from sources other than the nurse and peer educator was out of the researcher's control and of the study's limitations. Additionally, due to the lack of time, the obvious anxiety was only investigated in our study. Hence, according to the possibility of a difference in the levels of hidden anxiety, another study is suggested for the investigation and comparison of obvious and hidden anxiety. There is also a need for further studies to examine the effect of individual education by nurses and peers before the operation and even the effect of the length of treatment on anxiety as well as the medical consequences facing this group of patients.

## 5. Conclusion

The results of this study indicate the effect of nurse and peer education on preoperative anxiety in women candidates for ovarian puncture. Therefore, peer education is recommended when we are faced with the nurse staffing shortage.

##  Conflict of Interest

The authors declare that they have no conflict of interest in this research.
