# Corrigendum to “Comparing the effects of nursing versus peer-based education methods on the preoperative anxiety in infertile women: An RCT” [Int J Reprod BioMed 2019; 17: 883-890]

**DOI:** 10.18502/ijrm.v19i11.9919

**Published:** 2021-12-13

**Authors:** Farahnaz Farnia, Abbas Aflatoonian, Athareh Kalantari

In this issue of IJRM, Farahnaz Farnia and colleagues requested correction of their article titled “Comparing the effects of nursing versus peer-based education methods on the preoperative anxiety in infertile women: An RCT”. The authors reviewed the data and confirmed that critical but inadvertent typographical errors had occurred in the paper. As the authors explain in their letter to the editor, the errors are listed as:

• The word “randomized" has been changed to “evaluated" in the Materials and Methods section of the abstract. Also, the type of study has been corrected as “randomized clinical trial".
• The trial registration code has been added at the end of the abstract section.
• In the Materials and Methods section in the main text, the time of study has been changed to “February to August 2017".
• The type of study has been corrected as “randomized clinical trial", and the randomization description has been modified.
• The name and code of the ethics committee have been revised.
• In the statistical analysis section, the Chi-square test was also used, which has been added.
• The first four sentences of the Results section have been changed to: “From a total of 240 women candidates for the ovarian puncture, 42 women were excluded due to not meeting the inclusion criteria or unwillingness to participate during the intervention."
• Information about nurse education and control groups was incorrectly listed in table I, which was corrected. The total column has been deleted, and the ANOVA test at the bottom of the table has been changed to the Chi-Square test.
• The total row has been deleted in table II.

The analysis was reconducted and a corrected article has been provided with corrections to the paper and relevant tables. The authors have confirmed that there are no other errors. The corrected article has been reviewed, and we have confirmed that the overall conclusion has not been changed as stated in the updated article available at: http://dx.doi.org/10.18502/ijrm.v17i12.5795 (updated on November 29, 2021).

